# Muscle Protein Synthesis in Response to Plant-Based Protein Isolates With and Without Added Leucine Versus Whey Protein in Young Men and Women

**DOI:** 10.1016/j.cdnut.2024.103769

**Published:** 2024-05-10

**Authors:** Changhyun Lim, Tom AH Janssen, Brad S Currier, Nelani Paramanantharajah, James McKendry, Sidney Abou Sawan, Stuart M Phillips

**Affiliations:** Department of Kinesiology, McMaster University, Hamilton, Ontario, Canada

**Keywords:** protein supplement, essential amino acid, skeletal muscle, anabolism

## Abstract

**Background:**

Plant-based protein supplements often contain lower amounts of leucine and other essential amino acids (EAAs), potentially making them less effective in stimulating muscle protein synthesis (MPS) than animal-based proteins. Combining plant proteins could improve the EAA profile and more effectively support MPS.

**Objectives:**

The aim of this study was to determine the effect of a novel plant-based blend protein (PBP), PBP with added leucine (PBP + Leu) to levels equivalent to whey protein isolate (WHEY) on aminoacidemia and MPS responses in young men and women. We hypothesized that PBP + Leu would stimulate MPS equivalent to WHEY, and both would be greater than PBP.

**Methods:**

We employed a randomized, double-blind, crossover study consisting of 3 separate study visits to compare PBP, PBP + Leu, and WHEY. To measure MPS response to ingestion of the supplements, a primed continuous infusion of L-[ring^13^C_6_] phenylalanine was administered for 8 h at each study visit. Skeletal muscle tissue and blood samples were collected to measure aminoacidemia and MPS.

**Results:**

All protein supplements increased mixed MPS above postabsorptive levels (*P* < 0.001). However, MPS increase following ingestion of PBP was less than that following ingestion of PBP + Leu (*P* = 0.002) and WHEY (*P* = 0.046). There were no differences in MPS between PBP + Leu and WHEY (*P* = 0.052).

**Conclusions:**

Consumption of PBP isolate with added leucine stimulated MPS to a similar extent as whey protein in young men and women. PBPs containing higher leucine content promote anabolism to a similar extent as animal-based proteins.

This study was registered at clinicaltrials.gov as NCT05139160.

## Introduction

Muscle mass accretion occurs when the net balance between muscle protein synthesis (MPS) and muscle protein breakdown is positive. MPS is the variable most responsive to protein intake and exercise and the locus of control for positive muscle protein balance in healthy individuals [1,2]. Dietary proteins provide essential amino acids (EAAs), which are indispensable components to building muscle proteins and directly stimulate MPS via molecular pathways [3]. The quality of dietary protein is evaluated by its digestibility and amino acid (AA) profile [4,5]. Compared to animal-based protein, most plant-based proteins have a lower digestibility and a relatively lower EAA content per serving, with a limiting amount of one or more EAAs [6].

Leucine is the key EAA that stimulates MPS via activation of the mechanistic target of rapamycin signaling pathway [3,7]. However, most plant proteins have a lower leucine content [8]. For example, ingestion of plant-based protein results in a lower anabolic response compared with ingestion of isonitrogenous animal-source proteins in young and older individuals [9,10]. Nonetheless, the interest in plant-based protein and a transition toward plant-based diets is increasing [11,12]. Thus, strategies to enhance the anabolic response to plant protein sources for muscle may be advantageous.

The isolated forms of plant-based proteins have a higher digestibility and protein density than the raw form of dietary plants [13]. Combining 2 different sources of plant protein isolate can improve EAA profiles because one protein source can compensate for lower EAA in the other protein source [14,15]. Pinckaers et al. [16] reported that the ingestion of a plant-based protein blend (wheat, corn, and pea) stimulated muscle protein synthetic response to a similar extent as an equivalent amount of milk protein. To offer a broader spectrum of plant-based protein options, it is worth exploring novel combinations of diverse plant sources.

We aimed to examine the effect of a plant-based blend protein isolate (pea and canola protein) with and without added leucine (PBP and PBP + Leu) on the muscle protein synthetic response in young men and women. Although pea protein is considered a relatively higher-quality protein in plant sources, it has a lower content of methionine [17]. On the other hand, canola protein isolate contains a substantial amount of sulfur-containing AAs (i.e., methionine and cysteine, ≥3 g/100 g), which is higher than in any other plant source [18]. We compared PBP to whey protein isolate (WHEY; i.e., high-quality animal-based protein) in a controlled randomized crossover design in healthy young men and women. We hypothesized that *1*) ingestion of PBP would increase the MPS rate above the postabsorptive state; *2*) the MPS response to PBP + Leu would be greater than that of PBP; and *3*) the MPS response to the PBP + Leu would be similar to an equivalent amount of WHEY with an equal leucine content.

## Methods

This study was approved by the Hamilton Integrated Research Ethics Board (HiREB: 14040) and conducted according to the guidelines of the Declaration of Helsinki. All subjects were informed about the purpose, experimental procedures, and possible risks of the study before written informed consent was obtained.

### Participants

Total 8 healthy and recreationally active young men (*n* = 4) and women (*n* = 4) were recruited for this study. All participants were between 18 and 30 y old and nonsmokers, had a BMI between 18 and 30 kg/m^2^, and had not been involved in any structured resistance exercise program for at least the previous 3 mo. Participants who were using corticosteroids, anabolic steroids (e.g., testosterone), anti-inflammatory drugs, or hormonal contraceptives and had any gastrointestinal, neurological, or cardiovascular diseases or a history of neuromuscular or muscle disease were excluded. This study was registered at clinicaltrials.gov as NCT05139160.

### Study design

This study was a blinded, randomized, double crossover design consisting of 3 study visits. On each study visit, a different protein supplement was ingested: WHEY, PBP, or PBP + Leu, which had a leucine content matched to that of the WHEY supplement. The screening visit consisted of measuring height (m), body mass (kg), and body composition (lean mass [kg] and fat mass [kg]) assessed using dual X-ray absorptiometry (GE-Lunar iDXA). The order of treatments, PBP, PBP + Leu, or WHEY, was randomly assigned, and only one of the supplements was provided on each study visit. The randomization treatment order was computer-generated (http://www.randomization.com), and all procedures and analyses were performed in a double-blinded manner. On the study visits, participants underwent a primed constant infusion of L-[ring-^13^C_6_] phenylalanine and muscle biopsies to measure MPS rates and blood draws to evaluate circulating AA concentrations over 5 h following the ingestion of 1 of 3 supplements providing 20 g of protein per study visit.

### Diet

Prior to the first study visit, the habitual dietary information of each participant was evaluated using a 3-d dietary log (2 weekdays, 1 weekend day). Participants were instructed to maintain their regular dietary intake and to refrain from any strenuous physical activity or alcohol consumption for 2 d prior to the study visit. The evening prior to each study visit, participants ingested a standardized meal (Heart-to-Home Meal). After ingestion of the provided standardized meal, participants started an overnight fast. To ensure the meal was ingested, at the start of each study visit, participants were asked if they had finished the provided standardized meal.

### Infusion protocol

**FIGURE 1:**
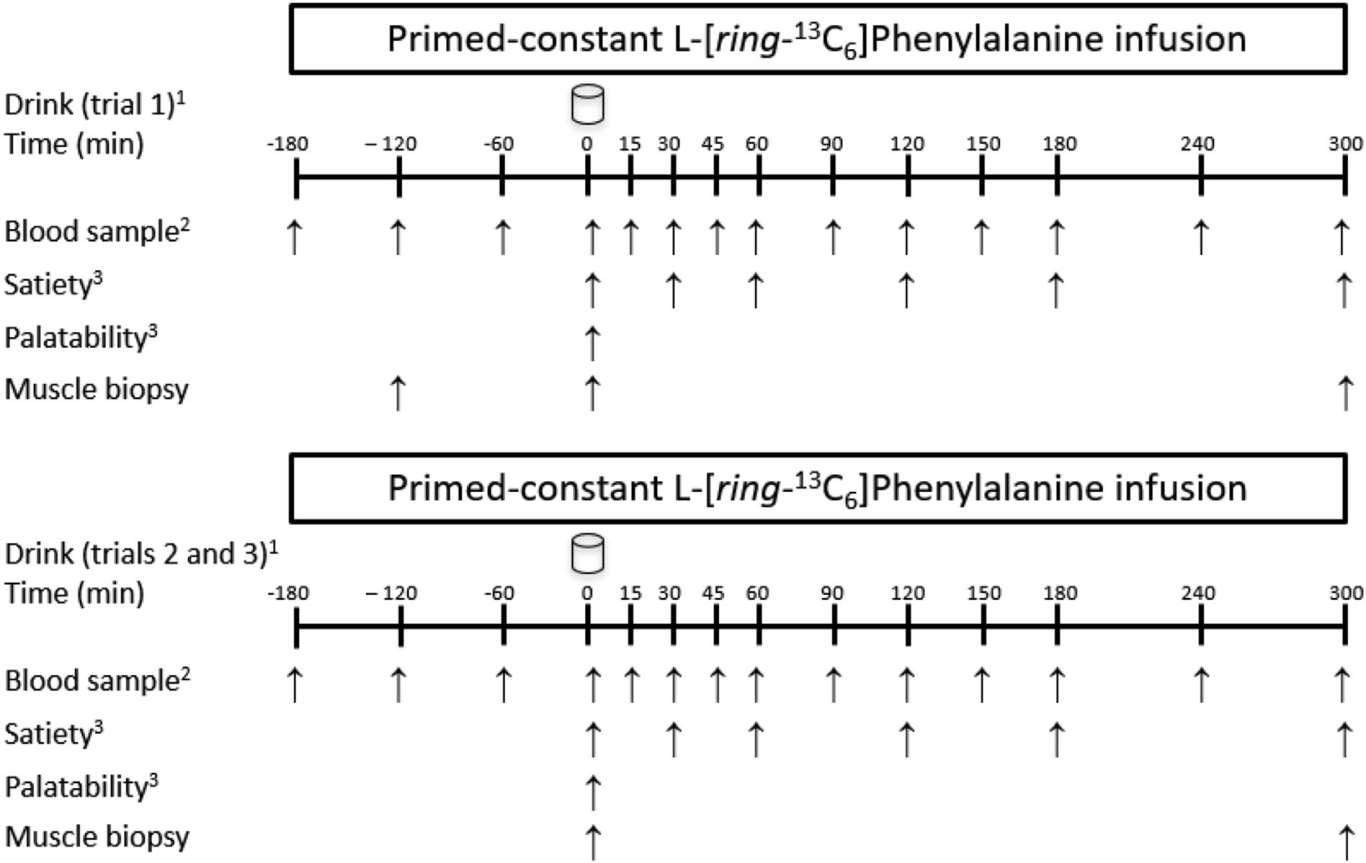
Schematic representation of the experimental protocol during the study visits. Negative and positive time points represent before and after drink consumption, respectively. ^1^Drink consumption (350 mL, the drinks contained 20 g of different protein isolates) were randomly assigned: PBP, PBP + Leu, or WHEY. ^2^To measure amino acid, insulin, and glucose concentrations, and plasma L-[ring-^13^C_6_] phenylalanine enrichment. Visual analog scales (1 to 10; Table 3). PBP, plant-based blend protein isolate; PBP + Leu, PBP fortified with leucine to match the leucine concentration in whey protein; WHEY, whey protein isolate.

A schematic overview of the study visits is presented in Figure 1. At the first study visit, subjects arrived at the laboratory at 07:30 following an overnight fast. First, a catheter was placed into the antecubital vein, and a heating pad was applied for 10 min prior to each given time to collect arterialized blood during the protocol. After the first basal blood collection, a second catheter was inserted in the contralateral arm for the infusion of L-[ring-^13^C_6_] phenylalanine. After a single intravenous priming dose of L-[ring-^13^C_6_] phenylalanine (2.0 μmol/kg) was administered (*t* = −180 min), the continuous intravenous infusion of L-[ring-^13^C_6_] phenylalanine (0.05 μmol/kg/min) was initiated using a calibrated pump (Harvard Apparatus). At −120 min, the first muscle biopsy sample was collected from the vastus lateralis of a leg using a 5-mm Bergstrom needle, custom-modified for manual suction, under local anesthesia (2% lidocaine). Blood samples were taken at −120, −60, and 0 min (Figure 1). Immediately following the collection of the second muscle biopsy sample from the same leg at 0 min, subjects ingested 1 of 3 protein supplements (i.e., PBP, PBP + Leu, WHEY), randomly assigned. Satiety and palatability questionnaires were obtained immediately following the ingestion of the supplement (*t* = 0 min) and at later time points (Figure 1). Blood samples were collected at the times indicated in Figure 1 after ingestion of the supplement. Following the collection of the last blood sample (*t* = 300 min), another muscle biopsy was collected. During the second and third study visits, the same procedures were repeated to evaluate the other 2 supplements, except for the first muscle biopsy at −120 min.

Blood samples were collected in EDTA-containing tubes and centrifuged at 1000 × *g* at 4°C for 10 min to obtain plasma. After centrifugation, the aliquots of the plasma were stored in a freezer at −80°C until further analysis. Any visible nonmuscle materials (e.g., connective tissue, blood, and fat) were removed from the collected muscle samples. The muscle samples were immediately frozen in liquid nitrogen and stored at −80°C until further analysis.

### Protein supplements

WHEY (SureProtein WPI 895) was obtained from Fonterra. PBP consisted of 88% pea protein (Peazazz) and 12% canola protein (Puratein), which were obtained from Merit Functional Food. The PBP + Leu leucine concentration was matched with that of WHEY and was mixed, taste-matched, and packaged by Gruppo Nutrition in sachets and shipped to McMaster without identifying marks but numbered. ∼25 g of the supplements (Table 1), which contained 20 g of protein, were dissolved in 350 mL water. The manufacturers provided the AA profiles of each supplement. The drinks were prepared by a blinded, independent assistant on the morning of the study visit. On the basis of phenylalanine content in the supplement, the drinks were enriched to 4% with L-[ring-^13^C_6_] phenylalanine to minimize disturbances in the isotopic steady state. Macronutrients and the specific AA compositions of the ingested supplements are presented in Table 1.

**TABLE 1:** Macronutrient and amino acid composition of the supplements

	PBP^1^	PBP + Leu^2^	WHEY^3^
Dosage (g)	23.2	24.7	21.3
Calories (kcal)	94.4	94.4	82.0
Protein (g)	20.0	20.0	20.0
Carbohydrate (g)	1.5	1.5	0.1
Fat (g)	0.9	0.9	0.2
Additional leucine (g)	—	1.5	—
EAA^4^ (g)			
Threonine	0.9	0.9	0.5
Valine	1.1	1.1	1.2
Isoleucine	0.9	0.9	1.3
Leucine^5^	1.5	3.0	3.0
Phenylalanine	1.0	1.0	0.8
Lysine	1.6	1.6	2.4
Histidine	0.5	0.5	0.4
Methionine	0.3	0.3	0.5
Tryptophan	0.2	0.2	0.5
Total	8.1	9.6	10.7
NEAA^6^ (g)			
Aspartic acid	2.3	2.3	0.6
Serine	1.0	1.0	1.0
Glutamic acid	3.8	3.8	3.7
Proline	1.0	1.0	1.0
Glycine	1.0	1.0	0.4
Alanine	1.0	1.0	1.2
Tyrosine	0.7	0.7	0.9
Arginine	1.6	1.6	0.6
Cysteine	0.3	0.3	0.9
Total	12.7	12.7	10.3

1 PBP, plant-based blend protein isolate; 88% pea protein and 12% canola protein.

2 PBP + Leu, PBP fortified with leucine to match the leucine concentration in WHEY.

3 WHEY, whey protein isolate.

4 EAA, essential amino acid.

5 Includes added leucine.

6 NEAA, nonessential amino acid.

### Plasma analysis

AAs were analyzed using the Waters Pico-Tag System (Waters). Blood samples were hydrolyzed, and precolumn derivatized using phenyl isothiocyanate (PITC) followed by reverse-phase high-performance liquid chromatography-mass spectrometry. In brief, samples were dried in pyrolyzed borosilicate tubes in a vacuum centrifugal concentrator and subjected to vapor phase hydrolysis by 6 N HCl with 1% phenol at 110°C for 24 h under a prepurified nitrogen atmosphere. After hydrolysis, excess HCl was removed by vacuum, and hydrolysates were washed with redrying solution and derivatized with PITC to produce phenylthiocarbamyl AAs. The derivatization method was accomplished at room temperature, derivatized both primary and secondary AAs (i.e., proline and hydroxyproline), and formed very stable AA derivatives detectable at low picomole levels. Derivatized AAs were redissolved in phosphate buffer and transferred to injection vials, which were loaded into the autosampler for automatic injection.

Plasma glucose concentrations were determined using a commercially available glucose monitor (Contour). In addition, plasma insulin concentrations were measured using a commercially available insulin ELISA kit (ALPCO, Human Insulin ELISA Kit). Incremental area under the curve (iAUC) was determined for plasma and insulin concentrations over the protocol using the trapezoid rule [19].

Plasma L-[ring-^13^C_6_] phenylalanine enrichments were measured using GC-MS (GC Hewlett Packard 6890; MS Agilent 5973) as previously described [2].

### Muscle analysis

Muscle protein-bound L-[ring-^13^C_6_] phenylalanine enrichments in whole muscle tissue (i.e., both sarcoplasm and myofibrillar fractions) were measured by GC-combustion-isotope ratio mass spectrometry (GC-C-IRMS) (GC Hewlett Packard 6890; IRMS model Delta Plus XP, Thermo Finnigan) to evaluate MPS rate in response to supplement ingestion, as previously described [2]. Briefly, muscle samples (∼30 mg) were homogenized in 1 M perchloric acid and then vortexed and centrifuged at 13,000 × *g* for 10 min at 4°C. After removing the supernatant, the remaining muscle pellets were washed with 70% ethanol and hydrolyzed with 1M HCl in the oven at 110°C for 72 h. The bound protein hydrolysate was passed over a cation-exchange column (Dowex 50WX8–200 resin, Sigma-Aldrich) and converted to their *N*-acetyl-n-propyl ester derivatives for analysis by GC-C-IRMS.

### Calculations

The MPS rate was calculated using the standard precursor-product method:

$$\text{MPS}\;\left(\%/h\right)=\left(\;\frac{\Delta E_{m}}{E_{p}\times t}\;\right)\times 100$$

$\Delta E_{m}$ represents the change in protein-bound L-[ring-^13^C_6_] phenylalanine enrichment between 2 samples. $E_{p}$ represents the average plasma-free L-[ring-^13^C_6_] phenylalanine enrichment during the tracer incorporation period, and t indicates the time interval (in hours) between biopsies.

### Palatability, hunger, and fullness questionnaire

Palatability, appearance, smell, taste, and aftertaste of the supplements were assessed immediately after ingestion of the supplements using a visual analog scale (VAS) in a double-blinded manner. The responses to the questionnaires were converted to a score of 1 to 10. A higher score represented a more favorable response.

Hunger and fullness were assessed at 0, 30, 60, 120, 180, and 300 min following ingestion of the supplements using VAS. iAUC was determined for hunger and fullness using the scores (1–10) converted from VAS; a higher score represented more hunger or more fullness.

### Statistics

A power calculation was performed based on previous research performed with the most similar methodological and intervention protocol as the present study to detect a difference in MPS. In the previous study, the postabsorptive levels of the MPS rate (0.025 %/h ± 0.06) were increased to 0.049 %/h ± 0.02 following ingestion of 25 g whey protein [2]. Therefore, with an α = 0.05, β = 0.8, and utilizing a within-participant comparison, 7 participants were determined to be sufficient to detect a significant difference in the primary outcome (i.e., MPS rate) between baseline and after supplement ingestion.

Data was assessed for normality using the Shapiro–Wilk test. Time-dependent variables (i.e., plasma glucose, insulin, AA concentrations, L-[ring-^13^C_6_] phenylalanine enrichments, and hunger and fullness scores) were analyzed using a 2-way repeated measures, within-subject analysis of variance (ANOVA), with time and treatment. MPS and iAUC of plasma glucose, insulin, hunger, and fullness were compared between treatments using a 1-way, within-subject ANOVA. The Tukey post hoc test was performed following the analysis of the significant interactions from 2-way ANOVA or differences from 1-way ANOVA. All data are presented as means ± SD. Statistical significance was set at *P* < 0.05. All statistical analyses were performed using R (version 4.3.2).

## Results

### Participant characteristics

A total of 8 (4 men and 4 women) young, healthy adults were recruited for this randomized crossover design study. All participants completed the study visits for PBP + Leu and WHEY, and 7 participants (4 men and 3 women) completed the study visit for PBP in a blinded manner. The participants’ characteristics are presented in Table 2.

**TABLE 2:** Baseline characteristics of participants

	Participants (*n* = 8)
Sex (M/W)	4M/4W
Age (y)	25 ± 4
Height (m)	1.75 ± 0.1
Body mass (kg)	79.5 ± 21.6
BMI (kg/m^2^)	25.5 ± 4.0
Lean mass (kg)	50.8 ± 14.9
Fat mass (kg)	25.1 ± 7.0
Body fat (%)	33.3 ± 4.4

Abbreviations: M, men; W, women.

Data are expressed as means ± standard deviation.

### Plasma glucose and insulin concentrations

**FIGURE 2:**
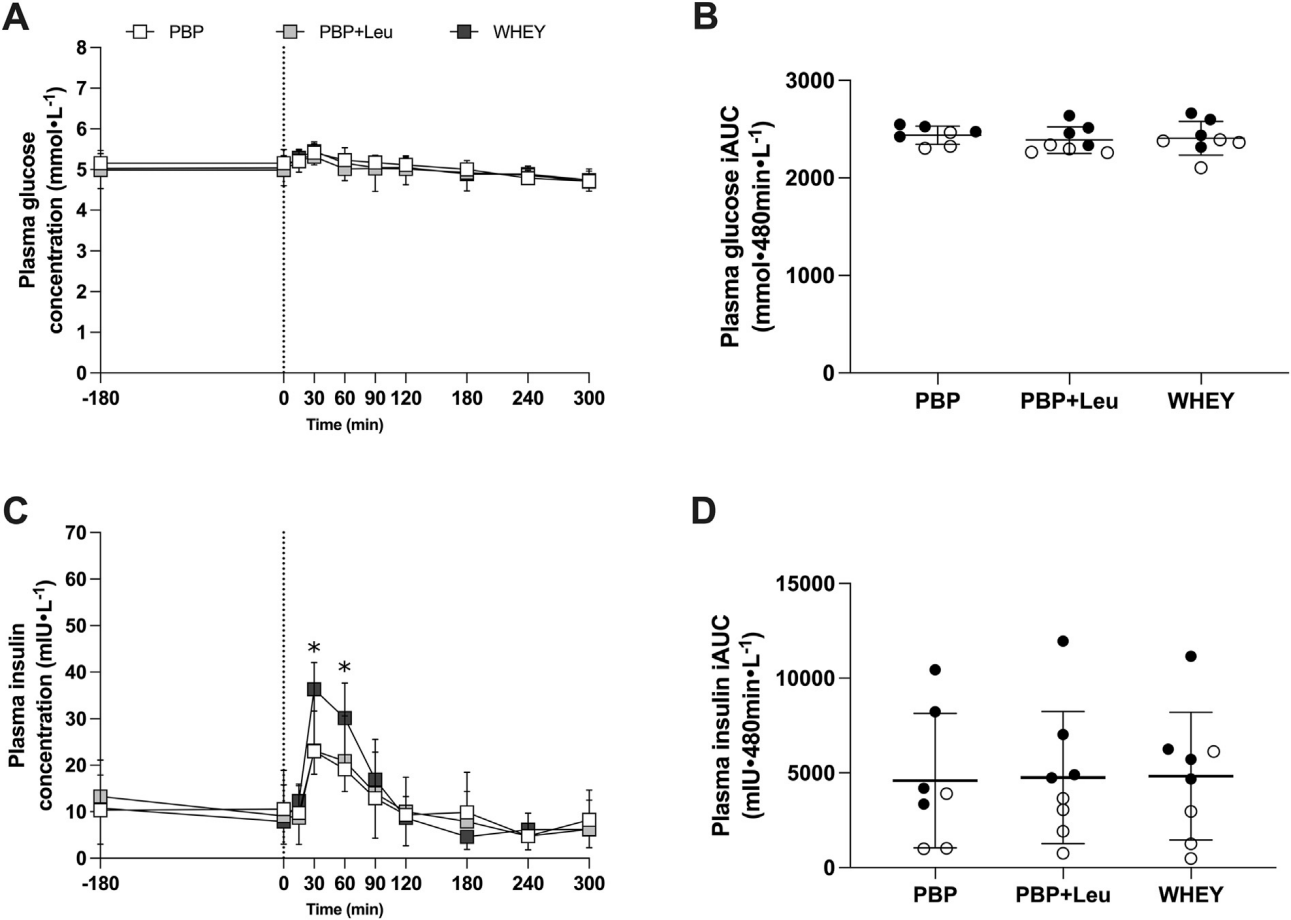
Plasma glucose (A) and insulin (C) concentrations during the protocol following ingestion of PBP (*n* = 7), PBP + Leu, or WHEY in healthy young men and women (*n* = 8). Panels (B) and (D) represent iAUC of glucose and insulin, respectively, following the ingestion of each treatment. 25 g of the supplements were consumed at 0 min. Data are expressed as means ± standard deviation. In panels (B) and (D), closed circles represent data from men, while open circles represent data from women. 2-way repeated measures ANOVA, within-subjects, was used to assess the changes in glucose and plasma concentrations. 1-way repeated measures ANOVA, within-subjects, was used to assess the iAUC. ANOVA, analysis of variance; iAUC, incremental area under the curve; PBP, plant-based blend protein isolate (88% pea protein and 12% canola protein); PBP + Leu, PBP fortified with leucine to match the leucine concentration in whey protein; WHEY, whey protein isolate.

Plasma glucose concentrations were increased at 30 min after protein ingestion with no difference among supplements (interaction, *P* = 0.984; treatment, *P* = 0.166; and time, *P* < 0.001) and returned to basal level by 60 min following protein ingestion (Figure 2A). There was no difference in iAUC in glucose concentration over time among protein supplements (*P* = 0.683; Figure 2B). There was an interaction between treatment and time in plasma insulin concentration (*P* = 0.013), and the Tukey post hoc test discovered that plasma insulin concentration following WHEY was higher than that following PBP and PBP + Leu at 30 (*P* < 0.001 and *P* < 0.01, respectively) and 60 min (*P* < 0.001 and *P* = 0.019, respectively; Figure 2C). There was no difference in iAUC in insulin concentration over time among protein supplements (*P* = 0.812; Figure 2D).

### Plasma AA concentrations

**FIGURE 3:**
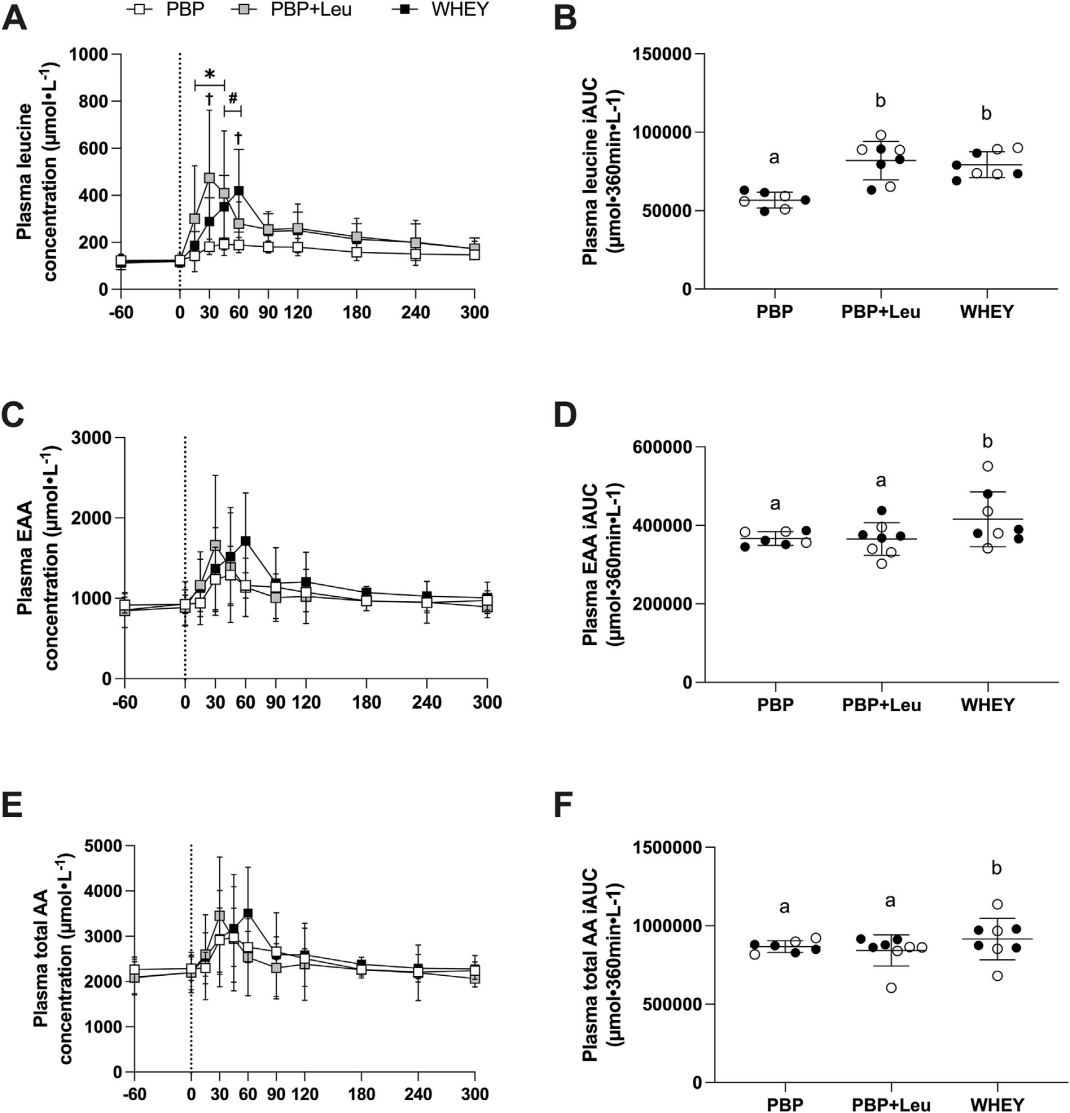
Plasma leucine (A), EAA (C), and total AA (E) concentrations during the protocol following ingestion of PBP (*n* = 7), PBP + Leu, or WHEY in healthy young men and women (*n* = 8). Panels (B), (D), and (F) represent the iAUC of leucine, EAAs, and total AAs, respectively, following the ingestion of each treatment. Supplements (25 g) were consumed at 0 min. Data are expressed as means ± standard deviation. In panels (B), (D), and (F), closed circles represent data from men, while open circles represent data from women. ∗*P* < 0.05, significant difference between PBP + Leu and PBP; #*P* < 0.05, significant difference between WHEY and PBP; †*P* < 0.05, significant difference between PBP + Leu and WHEY. 2-way repeated measures ANOVA, within-subject, was used to assess the changes in leucine, EAA, and total AA concentrations. 1-way repeated measures ANOVA, within-subject, was used to assess the iAUC. Means with different letters are significantly different. AA, amino acid; ANOVA, analysis of variance; EAA, essential amino acid; iAUC, incremental area under the curve; PBP, plant-based protein isolate (88% pea protein and 12% canola protein); PBP + Leu, PBP fortified with leucine to match the leucine concentration in whey protein; WHEY, whey protein isolate.

There was an interaction between treatment and time in plasma leucine concentration (interaction, *P* = 0.002; treatment, *P* < 0.001; time, *P* < 0.001; Figure 3A). Plasma leucine concentration was increased at 15, 30, and 45 min following PBP + Leu ingestion (all, *P* < 0.05) and at 30, 45, and 60 min following WHEY ingestion (all, *P* < 0.05). Plasma concentrations at 15, 30, and 45 min following PBP + Leu ingestion were 60% ± 47% (*P* = 0.011), 108% ± 54% (*P* < 0.001), and 132% ± 127% (*P* < 0.001) greater than those following PBP ingestion. Plasma concentrations at 45 and 60 min following WHEY ingestion were 75% ± 92% (*P* < 0.012), and 75.2% ± 108.1% (*P* < 0.001) greater than that following PBP ingestion. The peak plasma leucine was reached earlier following PBP + Leu ingestion (at 30 min) compared with WHEY (at 60 min). The plasma leucine iAUCs in PBP + Leu and WHEY were greater than that in PBP by 42% ± 17% (*P* < 0.001) and 41.7% ± 14% (*P* < 0.001), respectively (Figure 3B).

There was no interaction between treatment and time in plasma EAA (interaction, *P* = 0.350; Figure 3E). However, the main effects of treatment and time were detected in plasma EAA (treatment, *P* = 0.009; time, *P* < 0.001; Figure 3C). A pairwise comparison of the main effect of the supplements detected a difference in plasma EAA availability during the protocol between PBP and WHEY ingestion (*P* = 0.008). The plasma EAA iAUC in WHEY ingestion was 16% ± 14% (*P* = 0.019) and 14% ± 15% (*P* = 0.028) greater than that of PBP and PBP + Leu, respectively (Figure 3D).

There was no interaction between treatment and time in plasma total AA (interaction, *P* = 0.561; Figure 3E). However, the main effect of time was detected in plasma total AA (treatment, *P* = 0.068; time, *P* < 0.001; Figure 3E). The plasma total AA iAUC in WHEY ingestion was 9% ± 9% (*P* = 0.02) and 8% ± 11% (*P* = 0.028) greater than that of PBP and PBP + Leu, respectively (Figure 3F).

### MPS rate and plasma L-[ring-^13^C_6_] phenylalanine enrichment

**FIGURE 4:**
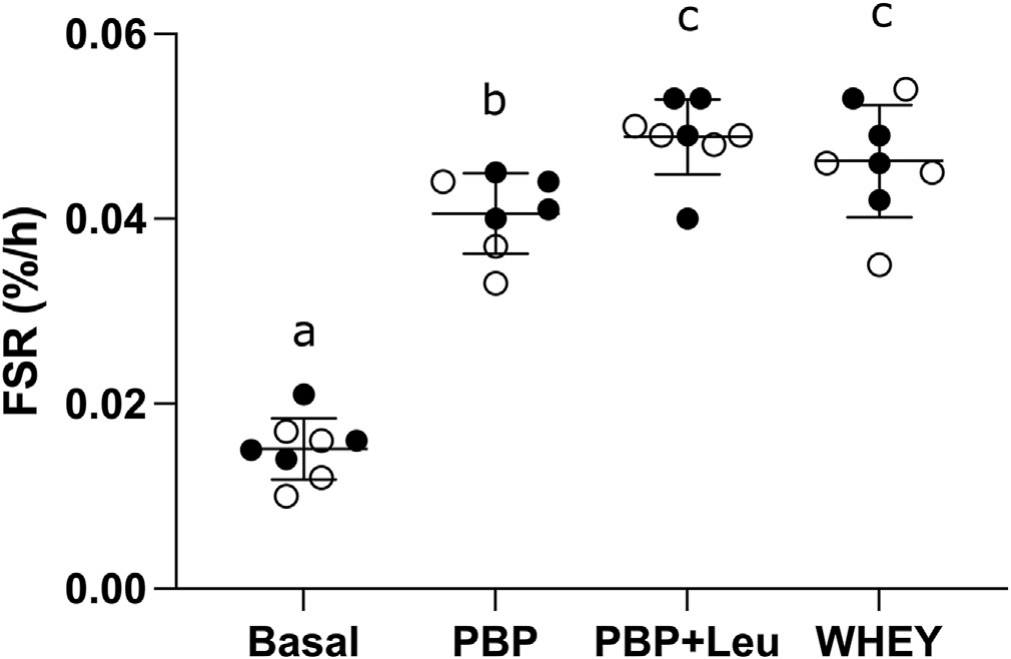
Mixed muscle fractional synthesis rate (%/h) following ingestion of the PBP (n = 7), PBP + Leu, or WHEY in healthy young men and women (n = 8). Data are expressed as means ± standard deviation (closed circles, men; open circles, women). 1-way repeated measures ANOVA, within-subjects, was used to assess the mixed FSR. Means with different letters are significantly different. ANOVA, analysis of variance; FSR, fractional synthesis rate; PBP, plant-based blend protein isolate (88% pea protein and 12% canola protein); PBP + Leu, PBP fortified with leucine to match the leucine concentration in whey protein; WHEY, whey protein isolate.

Compared to postabsorptive MPS levels (0.015 ± 0.003%/h), MPS measured over 5 h following ingestion of PBP, PBP + Leu, or WHEY was increased to 0.041 ± 0.004, 0.049 ± 0.004, or 0.046 ± 0.006 %/h, respectively (all, *P* < 0.001; Figure 4). There were no differences in MPS between PBP + Leu and WHEY (*P* = 0.052). However, the increased MPS following ingestion of PBP + Leu and WHEY was greater than that following the ingestion of PBP by 16.2% ± 11.6% (*P* = 0.002) and 12.1% ± 16.1% (*P* = 0.046), respectively.

There was no difference or change in plasma L-[ring-^13^C_6_] phenylalanine enrichment over time (interaction, *P* = 0.793; treatment, *P* = 0.145; time, *P* = 0.244; Supplemental Figure 1). The mean plasma L-[ring-^13^C_6_] phenylalanine enrichments over time were 6.4 ± 0.7, 6.4 ± 1.0, and 6.7 ± 0.8% (tracer/tracee) for PBP, PBP + Leu, and WHEY, respectively.

### Palatability, hunger, and fullness

There were no differences in appearance (*P* = 0.866), smell (*P* = 0.342), taste (*P* = 0.932), aftertaste (*P* = 0.824), and palatability (*P* = 1.0) between PBP, PBP + Leu, and WHEY (Table 3).

**TABLE 3:** Palatability questionnaires^1^

	PBP^2^	PBP + Leu^3^	WHEY^4^	*P*^5^
Appearance	7 ± 2.4	6.9 ± 2.9	6.6 ± 2.0	0.866
Smell	7 ± 1.6	7.3 ± 1.6	6.1 ± 2.5	0.342
Taste	4.4 ± 2.6	4.4 ± 1.4	4.6 ± 2.1	0.932
Aftertaste	4.5 ± 2.5	5.1 ± 1.4	4.4 ± 3.3	0.824
Palatability	5.9 ± 2.9	5.9 ± 2.3	5.9 ± 3.0	1.0

1 Visual analog scale (1–10) was used for the questionnaires. Data are expressed as means ± standard deviation.

2 PBP, plant-based blend protein isolate; 88% pea protein and 12% canola protein (*n* = 7).

3 PBP + Leu, PBP fortified with leucine to match the leucine concentration as whey protein (*n* = 8).

4 WHEY, whey protein isolate (*n* = 8).

5 1-way repeated measures analysis of variance, within-subjects, was used.

Hunger and fullness scores over time are shown in Supplemental Figure 2. There was no interaction between the effects of time and treatment in scores for hunger (interaction, *P* = 0.618) and fullness (interaction, *P* = 0.606). However, the main effect of time was detected in both the hunger score (treatment, *P* = 0.082; time, *P* < 0.001) and fullness score (treatment, *P* = 0.268; time, *P* < 0.001). There were no differences in iAUC in hunger (*P* = 0.082) and fullness scores (*P* = 0.702) over time between protein supplements.

## Discussion

The present study demonstrates that the consumption of PBP + Leu stimulated MPS to a similar extent as WHEY in young men and women. The ingestion of PBP without added leucine still increased MPS above postabsorptive levels but to a lesser extent than PBP + Leu or WHEY. While statistically different from WHEY and PBP + Leu, the stimulation of MPS by PBP was still relatively robust, considering a numerically small difference in the MPS compared with WHEY.

Compared with animal proteins, plant-based proteins often contain lower amounts of EAAs and may be limited in their ability to supply adequate EAAs due to lower levels of leucine, lysine, or sulfur-containing AAs [8]. Combining >2 different sources of plant-based protein could be an effective solution to improve EAA profiles by complementing the lower level of one or more AA. In this study, the PBP was a combination of 2 plant-based protein sources: pea protein isolate, which contains a moderate amount of leucine and lysine but low amounts of methionine; and canola protein isolate, which contains higher amounts of methionine to improve the EAA profile [8]. Consumption of PBP led to an increase in plasma EAA concentrations and stimulated MPS above postabsorptive levels, which is in line with a previous study conducted in our laboratory that showed an increase in MPS following the ingestion of potato protein when compared with fasting MPS [20]. However, the increase in MPS following ingestion of PBP was still lower than that following ingestion of WHEY, but only by 12% ± 16%. These results parallel previous studies showing plant-based protein isolates, such as soy or wheat, result in less stimulation of MPS, coincident with lower AA availability, compared with milk protein [6,21]. Nonetheless, the difference in MPS between PBP and PBP + Leu and WHEY was numerically small, and the physiological benefits would, we propose, be difficult to establish.

Despite no difference in total AA and EAA concentrations when subjects ingested PBP and PBP + Leu, PBP + Leu stimulated MPS more than PBP. Additionally, PBP + Leu comparably increased MPS to an extent no different than that of WHEY, along with a similar elevation in plasma leucine concentration. These data show that when all EAAs are supplied, the leucine content of the protein and subsequent leucinemia determines the muscle anabolic response. Several previous studies have already reported the role of leucine as an effective anabolic stimulator [22,23], and the present study supports the leucine “threshold” hypothesis of how protein ingestion induces MPS [24]. Furthermore, these data are supported by a previous study showing that a plant-based protein blend matched in EAA content, particularly leucine, to whey protein reached an equivalent MPS as the whey protein, even though the plasma total EAA concentrations and the plasma leucine concentrations were lower in the plant-based protein group compared with the whey group [25].

Based on the results not only from the current study but also from previous studies assessing the impact of plant-based protein [16,20,25], it seems that ingesting plant-based proteins is not a limited variable to induce anabolism in skeletal muscle, resulting in similar anabolic response as ingesting animal-based protein, as long as a sufficient amount of protein and all EAAs are consumed. However, it should be noted that this study specifically focused on young, healthy adults. Thus, older individuals who have a lower anabolic response to protein ingestion may respond differently to plant-based proteins compared to young adults. Although we predict that plant-based proteins with sufficient leucine content may induce effective anabolism as an alternative protein source in older adults, further study is needed in older individuals.

PBP and the PBP + Leu did not show any differences in palatability, fullness, and hunger compared to WHEY. These data are in line with the study conducted by Melson et al. [26], who showed no differences in hunger, fullness, satiety, desire to eat, and prospective food consumption following the ingestion of a liquid soy-based breakfast and a liquid whey-based breakfast.

The current study is the first to determine the effect of a PBP on MPS using a within-subject design, which improves statistical power and minimizes between-subject variability. A potential limitation of the current study is that we did not directly analyze the AA content of the supplements used in the current study and relied on the information provided by the manufacturer. However, the plasma EAA concentration following ingestion of the supplements was in line with the provided AA profiles. Additionally, we used the plasma phenylalanine pool to calculate MPS as a surrogate for the true precursor, the intracellular aminoacyl-tRNA pool, due to the technical difficulty of accurately measuring aminoacyl-tRNA. However, utilizing a blood precursor pool to measure MPS has been validated previously [27], and plasma and intracellular tracer enrichment, while giving relatively similar MPS trends [28]. For future directions, considering individual preference or accessibility to plant sources, novel combinations of diverse plant sources that generate higher leucine content with all EAAs are required to provide a wide range of plant-based protein options and individualized alternatives to animal-based protein for MPS improvement. Additionally, determining the efficacy of PBPs in clinical populations or older adults or conducting longer-term studies investigating the effect of PBPs will provide valuable insights into the application of plant-based protein ingestion.

We conclude that the ingestion of 20 g of pea and canola protein isolate blend was able to increase MPS above postabsorptive levels but not as effectively as whey protein; however, when leucine content was matched, the pea and canola protein isolate blend stimulated MPS to the same degree as whey protein in healthy young adults. Thus, the present study shows the efficacy of plant-based protein containing higher leucine content in promoting anabolism as an alternative to animal-based protein.

## Author contributions

The authors’ responsibilities were as follows – CL, TAHJ, BSC, SAS, SMP: designed research; CL, TAHJ, BSC, NP, JM: conducted research; CL, TAHJ, BSC, SMP: analyzed data; CL, TAHJ, BSC, NP, JM, SAS, SMP: wrote article; CL, TAHJ, SMP: had primary responsibility for the final content; and all authors: read and approved the final manuscript.

## Funding

This work was supported by funding from the Ontario Center of Innovation (OCI). The sponsor provided interventional products, but the sponsor had no role in study design, data collection, and analysis and no such involvement or restrictions regarding publication. JM and CL were supported by postdoctoral fellowships from the Canadian Institute of Health Research (CIHR). TAHJ was supported by MITACS. BSC was supported by a Canadian Graduate Scholarship from the Natural Sciences and Engineering Research Council of Canada (NSERC). The funders had no role in the study design, collection, analyses, or interpretation of data, in the writing of the manuscript, or in the decision to publish the results.

## Data availability

Data described in the manuscript will be made available upon request pending.

## Conflict of interest

SMP reports grants or research contracts from the US National Dairy Council, Canadian Institutes for Health Research, Cargill, Friesland Campina, Dairy Farmers of Canada, Roquette Freres, Ontario Center of Innovation, Nestle Health Sciences, National Science and Engineering Research Council, and the US NIH during the conduct of the study; personal fees from Nestle Health Sciences; and nonfinancial support from Enhanced Recovery, outside the submitted work. SMP holds patents licensed to Exerkine but reports no financial gains from patents or related work. All other authors report no conflicts of interest.
