# Supplementary material for: Muscle Protein Synthesis in Response to Plant-Based Protein Isolates With and Without Added Leucine Versus Whey Protein in Young Men and Women
Source: Curr Dev Nutr. 2024 May 10;8(6):103769. doi: 10.1016/j.cdnut.2024.103769 (PMC11153912; doi:10.1016/j.cdnut.2024.103769)
Supplement: Multimedia component 1 [file mmc1.docx]

**Supplementary Data (2 figures)**

**Supplementary Figure 1.** Plasma L-[ring-^13^C_6_] phenylalanine enrichments following ingestion of the PBP (n = 7), PBP+Leu, or WHEY in healthy young men and women (n = 8). PBP, plant-based blend protein isolate (88% of pea protein and 12% of canola protein); PBP+Leu, PBP fortified with leucine to match the leucine concentration with whey protein; WHEY, whey protein isolate; Data are expressed as means ± standard deviation. Two-way repeated measures ANOVA, within-subjects, was used.

**Supplementary Figure 2**. Hunger (A and B) and fullness (C and D) over the protocol following ingestion of the PBP (n = 7), PBP+Leu, or WHEY in healthy young men and women (n = 8). PBP, plant-based blend protein isolate (88% of pea protein and 12% of canola protein); PBP+Leu, PBP fortified with leucine to match the leucine concentration with whey protein; WHEY, whey protein isolate; iAUC, incremental area under the curve. 25 g of the supplements were consumed at 0 min, and the MPS was measured over 5 hours following ingestion of the supplements. Data are expressed as means ± standard deviation. Two-way repeated measures ANOVA, within-subjects, was used to assess the changes in hunger and fullness scores. One-way repeated measures ANOVA, within-subjects, was used to assess the iAUC, incremental area under the curve, of them. There was no difference in pairwise comparisons using the Tukey post-hoc test (*P* > 0.05).
